# The Potential of Pharmaceutical Hydrogels in the Formulation of Topical Administration Hormone Drugs

**DOI:** 10.3390/polym14163307

**Published:** 2022-08-14

**Authors:** Aneta Ostróżka-Cieślik

**Affiliations:** Department of Pharmaceutical Technology, Faculty of Pharmaceutical Sciences in Sosnowiec, Medical University of Silesia, Kasztanowa 3, 41-200 Sosnowiec, Poland; aostrozka@sum.edu.pl

**Keywords:** hydrogels, hormone, topical, permeation enhancers

## Abstract

Hormones have attracted considerable interest in recent years due to their potential use in treatment of many diseases. Their ability to have a multidirectional effect leads to searching for new and increasingly effective drugs and therapies. Limitations in formulating drug forms containing hormones are mainly due to their low enzymatic stability, short half-life and limited bioavailability. One of the solutions may be to develop a hydrogel as a potential hormone carrier, for epidermal and transdermal application. This review discusses the main research directions in developing this drug formulation. The factors determining the action of hormones as drugs are presented. An analysis of hydrogel substrates and permeation enhancers that have the potential to enhance the efficacy of hormones applied to the skin is reviewed.

## 1. Introduction

In recent times, a great deal of attention has been given to hormones as medicines. They show high efficacy by binding to specific receptors and causing few off-target side-effects [1]. Hormone therapy is most often carried out to compensate for endogenous hormone deficiencies or to achieve a pharmacological effect in the case of diseases not caused by hormone disorders [2,3]. In the first case, hormone replacement therapy, which involves the use of native hormones, is used. Hormones obtained by isolation from tissues or hormones modified by biotechnological methods are used [4,5]. The dose of hormone used usually does not exceed physiological concentrations in blood serum. On the other hand, in order to obtain a specific pharmacological effect, doses exceeding physiological ones are mostly used, and hormones are often replaced by suitably modified molecules [6]. The potential pharmacological action of hormones is still the subject of intensive research [7,8]. For example, recent literature reports confirm the effectiveness of growth hormone therapy in fibrosis, cardiovascular diseases, cancer and nonalcoholic fatty liver disease [9,10]. In turn, Pan et al. [11] suggest therapeutic potential of melatonin in colorectal cancer.

In parallel, research is being conducted to develop forms of biopharmaceuticals. Limitations to their use are due to their high clearance, poor membrane permeability, negligible activity when administered orally, sometimes poor solubility and their high manufacturing cost [1]. The therapeutic activity of hormones depends largely on their complex structure, which must be stable during the formulation of the drug form and during the release at the site of action. Technological processes must be selected in such a way that the structure of hormones is not altered (change in the structure of hormones weakens their action or even lead to the formation of immunogenic structures) [12,13]. To ensure optimal stability of hormone raw materials, they are stored frozen or in freeze-dried form with the addition of appropriate auxiliary substances [14,15,16,17].

## 2. Factors Determining the Action of Hormones as Drugs

The most significant technological problem in formulating drug forms containing hormones is the limited possibility of transporting the active substance to its site of action. Absorption of proteins and peptides after oral administration is usually impossible with traditional forms of drug administration such as tablets and capsules. This is due to a lack of membrane permeability, low oral bioavailability and low metabolic stability [1,18]. Proteolytic enzymes are present in the gastrointestinal tract, leading to the breakdown of proteins and peptides. In the intestinal mucosa, API metabolism occurs, mainly under the influence of cytochrome P450 (CYP) isoenzyme 3A4 and *p*-glycoprotein. In addition, the mucosa of the gastrointestinal tract is a resorption barrier for protein hormones, which are water-soluble [19,20,21]. For most hormonal preparations, the application route is parenteral administration, including intravenous, intramuscular, subcutaneous, transdermal, vaginal, rectal, sublingual, intranasal and inhalation [22]. However, it should be borne in mind that proteolytic enzymes are distributed throughout the body (in the blood, vascular bed, on cell membranes, and inside cells) and determine the specific metabolic pathway of a given organ [23]. Therefore, the bioavailability of hormones after parenteral administration may vary, even a little.

**Figure 1:**
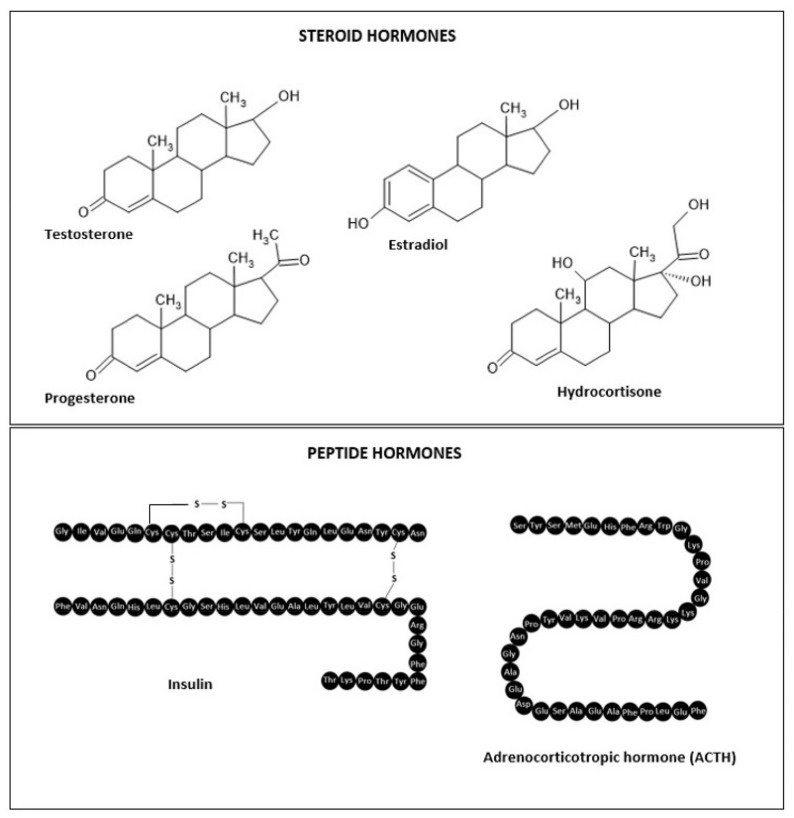
Chemical structures of hormones (created with ACD/ChemSketch software 2020.2.1, Advanced Chemistry Development, Inc., Toronto, ON, Canada).

The effect of the applied hormone depends on many factors. One of them is the stage of binding of the hormone by plasma proteins. The biologically active form of the hormone is the free fraction of the hormone (this is especially true for thyroid hormones and steroid hormones) [24]. Another determinant of hormone activity is metabolism, which can lead to the inactivation of the API (Active Pharmaceutical Ingredient) or the formation of a biologically active form. An example is a testosterone, which is converted in tissues to the active metabolite dihydrotestosterone [25,26,27]. An essential determinant of hormone action is the presence of receptors. The greater the number of receptors, the greater the effectiveness of hormone therapy. Hormones are characterized by a short half-life, which is beneficial in the process of regulating their physiological action [28]. From the point of view of pharmacotherapy, this factor necessitates frequent administration of the drug. Therefore, efforts are being made to develop a form of the hormone that will ensure API bioavailability for longer periods of time, improved transport across biological membranes and higher stability and longer plasma half-lives [21]. A potentially effective drug formulation for the hormone could be a gel for administration on the skin. Most of the studies available in the literature are concerned with optimizing the formulation of this carrier to increase the penetration of the hormone through the skin (Table 1). Figure 1 shows the chemical structures of the tested hormones. For example, a commercially available formulation of Androgel^®^ provides a bioavailability of testosterone of 10% when administered to the skin [22,25,29,30].

## 3. Factors Determining the Effect of Hormones Applied to the Skin

The skin represents an alternative route of administration of the hormone to oral and parenteral administration, e.g., by injection. The advantages of this route of API administration are the high safety of use and efficacy for wound healing [48]. The first-liver-pass effect is avoided. Decomposition of the active substance in the gastrointestinal tract is eliminated, and interactions with food and orally administered drugs are avoided. The occurrence of adverse reactions is limited. The application of the drug is simple and does not require the participation of medical personnel [49,50,51]. Protein and peptide molecules pose a challenge in formulating a stable dermatological drug formulation. An additional difficulty is that they must overcome the barrier that is the intercellular lipid bilayer in the stratum corneum. Several methods are currently used to increase skin penetration e.g., pro-drugs, iontophoresis, phonophoresis, electroporation, heat enhancement and micro-needle systems. However, the most frequently used are modifications of the properties of the horny layer of the skin (occlusion, permeation enhancers). These methods allow for increasing the range of transdermally applied biologically active substances and searching for new areas of their pharmacological action [30,51,52].

The penetration and absorption of API can occur in several ways. The active substance may accumulate at the skin surface, in the stratum corneum. The API can be absorbed by passive diffusion into the epidermis or penetrate the dermis (penetration). The biologically active substance may also penetrate the subcutaneous layer (where blood vessels are located) by resorption [53,54].

One of the factors limiting the transdermal administration of API is the lipophilicity of the stratum corneum (the outermost layer of the skin). It has been found that this layer is penetrable mainly by lipophilic substances (log *p* = 1–3), non-polar and of molecular weight <500 Da. It is impermeable for peptides and polypeptides with molecular weight >1000 Da [55,56,57]. Molecules with a branched structure are also more difficult to penetrate. Lipophilic compounds penetrate tissue structures and bind to the intercellular binder of the stratum corneum, making it difficult for them to penetrate deeper into the skin. Hydrophilic molecules, on the other hand, have the ability to penetrate moistened skin and penetrate deeper into the skin [58,59]. Substances of lipophilic character more easily diffuse from a hydrophilic substrate, whereas substances of hydrophilic character more easily diffuse from a lipophilic substrate. The fastest permeation through the skin is saturated solutions of API. It has been found that the rate of permeation increases with increasing concentration. The pH value of the preparation should be between 5 and 10 to avoid skin irritation after application. The skin pH value is approximately 5 [60,61].

A factor influencing the penetration of hormones is the skin’s metabolism, although it is small. In the epidermis, there are enzyme systems capable of metabolising biologically active substances. Cytochrome P450 enzymes, epoxide hydrolase, N-acetyl transferases, glucuronyl transferases and sulfatases are present. The cutaneous activity of CYP 450 enzymes is 1–5% of the hepatic activity, while transferases are about 10% [62,63]. This affects the concentration of the biologically active substance in the skin. Therefore, it is necessary to ensure a sufficiently long residence time of the gel on the skin.

The skin is a hormone-dependent organ in which receptors, mainly sex hormones, are present [64]. Receptors can be located in the cell membrane (membrane receptors), the cytoplasm of the cell (cytosolic receptors) and in the chromatin of the cell nucleus (nuclear receptors) [65,66,67,68]. Peptide hormones (e.g., insulin, anterior pituitary hormones) are lipophobic (good water solubility and short half-life) and are not able to penetrate the cell membrane directly [69]. They bind to surface membrane receptors and pass into the cell. Their transport takes place with the help of extracellular fluid [70]. Steroid hormones (e.g., androgens, estrogens, progestogens) are lipophilic in nature and are able to penetrate the double lipid layer of the cell membrane [71]. They bind to specific receptors in the cytoplasm or in the cell nucleus [72,73]. For example, the estrogen receptors ERα (encoded on chromosome 6) are located in fibroblasts and macrophages. Estrogen receptors Erβ (encoded on chromosome 14) are found in melanocytes, keratinocytes of the basal layer of the epidermis, dendritic cells and vascular endothelium. Androgen receptors (AR) and the enzyme 5-α-reductase, which is involved in the conversion of testosterone to DHT, are found in the basal layer of differentiated sebocytes, epidermal keratinocytes and fibroblasts. Glucocorticosteroid receptors are expressed in keratinocytes of the basal layer of the epidermis and fibroblasts of the dermis [63,74,75,76]. Cortisol, adrenocorticotropin (ACTH) and corticotropin (CRH) receptors have been found in keratinocytes, melanocytes and skin fibroblasts [77]. Melatonin receptors are located in epidermal keratinocytes, dermal fibroblasts and vascular endothelium [78]. Prolactin (PRL) mRNA has been found in dermis fibroblasts [79]. Receptors for insulin are present in keratinocytes and epidermal fibroblasts [80].

The parameters determining the transdermal administration of biologically active substances are the condition of the skin (pathogen infection, lesions in the form of cracks, exudates, etc.), the physicochemical properties of the API (molecular weight, polarity and lipophilicity, i.e., optimal hydrophobic-hydrophilic properties, log *p* partition coefficient), the presence of promoters of transdermal transport and the type of carrier used (dermatological substrate). The higher the affinity of the active substance to the lipid layer of the epidermis, the greater its ability to penetrate the stratum corneum [39,53,81]. Furthermore, increasing the amount or volume of gel applied per cm^2^ of skin increases the permeation and absorption of the hormone [82].

## 4. Potential of Hydrogels as Carriers of Hormones Administered to the Skin

The type of carrier used significantly affects the efficacy of a dermatological drug. It allows for maintaining the optimal activity of the peptide or influencing its inactivation [83]. Carriers containing components with high affinity to the active substance should be avoided as this may result in a lack of API release into the skin [53,84]. The effective action of the drug is influenced by the physicochemical form of the vehicle. Recent years have seen the rapid development of hydrogels. Initially, they were used as implants and scaffolds in tissue engineering. Over time, they began to be used as carriers of biologically active substances [49]. This is due to their biocompatibility and the similarity of their physical properties to natural tissue [85]. First-generation hydrogels were based on chemical modification of monomer or polymer with an initiator. Second-generation hydrogels respond to specific stimuli like variations in temperature, pH, or concentration of molecules in the solution [86]. Hydrogels have been used in pharmaceutical technology for modified topical drug delivery for 60 years [87].

Gels may be lipophilic or hydrophilic in nature. Oleogels (lipophilic gels) are obtained by gelling lipophilic liquids (mineral oils, vegetable oils, synthetic oils) with cross-linking substances (colloidal silica, aluminum or zinc stearate) [88,89]. Hydrogels (hydrophilic gels) are solutions of polymers that form an internal, organised spatial structure (3D). Natural (e.g., agar, alginates) or synthetic polymers (e.g., cellulose derivatives, poloxamers, polyacrylates) are used to prepare hydrogels. The dispersion medium is water [90,91,92]. Auxiliary substances used in hydrogels are glycerol, propylene glycol and ethanol [93]. The spatial network of a hydrogel is formed by polymer chains that are connected by molecular forces, ionic interactions, hydrogen bonds or hydrophobic interactions. When choosing a polymer, attention should be paid to its effect on the pharmacokinetics of the active substance. For example, carbopoles can accelerate the release of weak acidic substances or slow down the release of weak basic API. On the other hand, weakly basic cellulose derivatives may cause hydrolysis of the active substance [92,94]. Table 2 shows the physicochemical parameters of the hormones and the hydrogel-based used for them.

Analyzing the compositions of commercial hydrogels containing hormones (Table 2) and those in the research phase (Table 1), the universal use of carbomers draws attention. Carbomers are acrylic acid polymers cross-linked with alkenyl ethers of sugars or polyalcohols, with a molecular weight from 700 kDa to 4,000,000 kDa. Carbomers are available under the trade name Carbopol. In the anhydrous state, the polymer molecules are coiled. When water is introduced, they hydrate and partially expand. Neutralisation of the carboxyl groups of the polymer (with sodium hydroxide or triethanolamine) causes the polymer chains to unwind into a linear structure and a gel is formed. The versatility of this polymer results, among other things, in the possibility to prepare a stable gel in both neutral and acidic media. The gelation process with glycerol or propylene glycol allows for the addition of acidic active substances to the carrier. Carbomer-based hydrogels do not adhere to wounds and accelerate their healing. Several types of carbomers are available on the market, which differs in the percentage of crosslinking agents used [29,94,103]. This affects the process of API release from these hydrogels.

Hydrogels prepared with cellulose derivatives were also used as hormone carriers (Table 1 and Table 2). Methylcellulose, sodium carmellose (sodium carboxymethylcellulose) and hydroxyethylcellulose hypromellose (hydroxymethyl propyl cellulose) were used to prepare hydrogels. The technique for preparing the carrier consists of dissolving the polymer in water (in water with glycerol or propylene glycol) and adding a solution of the active substance, with intensive stirring. Hydrogels based on cellulose ethers show high stability and biological neutrality [104,105]. A study by Heo et al. [32] confirmed the possibility of using a hydrogel based on hydroxypropyl methylcellulose as an effective testosterone carrier. In a preformulation study on the development of a hydrogel carrier for insulin, Ostróżka-Cieślik et al. [39] tested the efficacy of Carbopol Ultrez 10, Carbopol Ultrez 30, methylcellulose, glycerol ointment in an in vitro model. Methyl cellulose-based hydrogel released insulin reaching 75% after 9 h. The hormone was released gradually in a prolonged manner. The formulation exhibited optimal physicochemical properties, facilitating its application to the skin.

An et al. [31] used polyvinyl alcohol (PVA) to obtain a hydrogel. The gel preparation technology involves dissolving the polymer at an elevated temperature followed by intense stirring until a transparent substrate is obtained. This hydrogel, when applied to the skin, forms a palpable membrane that prevents the drug from leaching out and releases the API over an extended period of time. The dried layer of hydrogel on the skin can last up to 24 h.

Chitosan is a natural cationic polysaccharide. It shows a beneficial effect on the absorption of active substances characterised by negligible permeability through biological membranes [106]. It is biocompatible, biodegradable and nontoxic. Chitosan-based hydrogels exhibit antimicrobial and hemostatic properties. It has been suggested that chitosan and its derivatives increase the permeation of API through the skin. It probably affects the barrier properties of the cellular junctions [107]. The presence of chitosan in epithelial cell cultures also increases fibroblast growth factor 7 (FGF7) and fibroblast growth factor 10 (FGF10) [108]. Kählig et al. [36] studied the effect of three types of hydrogel substrates on progesterone release. They compared chitosan-EDTA, carrageenan and chitosan-glycolic acid in a porcine abdominal skin model in vitro. Chitosan-EDTA shows high antimicrobial activity; however, the adhesiveness is too small. The presence of a chelating agent (EDTA) in the hydrogel leads to a prolonged drug release. Low chelating and cross-linking agent concentrations impact slow-release API [107]. The authors found that chitosan-glycolic acid showed increased adhesion to the skin and higher viscosity. The amount of progesterone released after 48 h of testing decreased in a series: chitosan-glycolic > carrageenan > chitosan-EDTA. In contrast, Meler et al. [46] compared the release of hydrocortisone (HC) from several carriers (methylcellulose, carboxymethylcellulose Carbopol 934P, chitosan) in the presence/absence of 10% propylene glycol-1,2. HC was released faster from cellulose-based substrates than Carbopol 934P. Chitosan in the system with cellulose derivatives affected the acceleration of HC release. Semiliberation rates of hydrocortisone from hydrogels were: t50% = 6.65 (3% methylcellulose, 10% propylene glycol-1,2, 1% chitosan), t50% = 5.64 (3% sodium carboxymethylcellulose, 10% propylene glycol-1,2, 1% chitosan), t50% = 5.11 (3% Carbopol 934, 10% propylene glycol-1,2, 1% chitosan), respectively.

Bassani and his team [38] incorporated progesterone into a ready-to-use VersaBase^®^ substrate. This hydrogel is resistant to low pH and shows compatibility with polar solvents. Studies were performed in a model of transdermal absorption of progesterone through human skin. In addition, 21.8% of the hormone was absorbed through the skin.

## 5. Permeation Enhancers That Promote the Penetration of Hormones through the Skin

The permeation of peptide substances through the skin can be increased by affecting the hydrolipid layer of the epidermal surface. For this purpose, inclusion dressings or substances that facilitate API penetration into deeper skin layers are used. Absorption promoters are chemical compounds that exhibit the ability to reversibly alter the structure of the epidermal lipid matrix (disordering or fluidizing effects on the stratum corneum/SC lipids) and allow diffusion of the active substance. They may also modify corneocyte proteins or affect the solubility and partition coefficient of API. The ideal API permeation enhancer should be pharmacologically inactive, non-irritating, non-toxic and non-damaging to the skin and stable. The most commonly used solubilizers are low molecular weight alcohols (ethanol, isopropanol, propylene glycol), esters of alcohols and fatty acids (isopropyl myristate, isopropyl palmitate) and terpenes and their derivatives [109,110]. Mixtures of some substances, e.g., isopropyl myristate (IPM) and alkanols (ethanol, glycerol, isopropanol, propylene glycol) show synergism of action [111].

### 5.1. Fatty Acids and Surfactants

Fatty acids are often used to improve the transdermal delivery of hormones (estradiol, progesterone). Their action involves modification and disruption of the lipid matrix in the stratum corneum. It is suggested that they show higher efficiency for the absorption of lipophilic drugs. The beneficial features of fatty acids include the non-irritational effect on the skin, no toxicity, wide range of compatibility, high skin flux, reduced skin irritation and sensitization [112]. A synergistic effect of the system was found in propylene glycol-lauric acid w transdermal delivery of highly lipophilic drugs (antiestrogens AE 1/log *p* = 5.82 and AE 2/log *p* = 7.8) [113]. Oleic acid is effective at low concentrations (10%) [114]. In higher concentrations, it can act as a separate phase within the bilayer lipids, thus facilitating the permeation of hydrophilic permeants through the membrane [115].

Surfactants are usually added to solubilise lipophilic active ingredients. They enable permeation of drugs via a transdermal route [116]. At low concentrations, they act by solubilising lipids within the stratum corneum, disrupting the lipid and protein domains. They can penetrate through the lipid bilayer. The ability of surfactants to penetrate the stratum corneum depends on the partitioning behavior and solubility. They exhibit hydrophobic (oleic acid) and hydrophilic (sodium lauryl sulfate) properties. Their action causes skin irritation. Hydrophilic surfactants are generally less irritating and better tolerated than poloxamer, poloxamine and polysorbates [117]. It has been suggested that surfactants have a more lipid disorientating effect in the stratum corneum and create higher levels of cutaneous absorption than terpenes, alcohols and glycols [118].

Ann et al. [31] developed polyvinyl alcohol with polyisobutylene-based hydrogel formulation for testosterone (TS). They analysed the effect of selected excipients, i.e., dodecylamine, 1-(2 (Decylthio)ethyl)azacyclopentan-2-one (HPE101), oleic acid and lauric acid, on the rate of hormone permeation through the skin. In vivo studies were conducted in a rat model, applying the hydrogel on the dorsal skin. On the other hand, in vitro studies were carried out with Keshara–Chien permeation cells, using a fragment of rat dorsal skin. The authors confirmed the high efficacy of dodecylamine at a concentration of 3%. In in vivo studies, the area under the curve (AUC24hr) values calculated from the plasma concentration profiles of TS increased from 77.73 ng*h/mL to 407.29 ng*h/mL. In vitro, the permeation rate of TS in the presence of 3% dodecylamine increased 10-fold (0.54 μg/cm^2^/h—without an absorption promoter, 4.92 μg/cm^2^/h in the presence of 3% dodecylamine). The addition of 5% dodecylamine to the hydrogel reduced the TS permeation rate, probably due to an increase in the viscosity of the substrate.

Barreiro-Iglesias et al. [43] investigated the potential of carbopol/surfactant dispersion in the controlled release of estradiol. The efficacy of Carbopol^®^ 934 was evaluated in the presence of Pluronic F-127, Tween 80, sodium dodecyl sulfate (SDS), and benzalkonium chloride (BkCl). Carbopol/surfactant aggregates increase the solubility of hydrophobic drugs. The authors suggest that, by choosing surfactants with desirable properties, e.g., with appropriate HLB (Hydrophilic Lipophilic Balance), one may modulate the strength of hydrophobic interactions between carrier components and control the rate of API release (also in low viscosity medium). Estradiol release mainly seems to happen as a direct exchange between the carbopol/surfactant aggregates and the surfactant micelles of the receptor medium. The lack of organic solvent in the proposed formulations and the acidic pH potentially avoid the occurrence of adverse reactions after skin application.

Matsui et al. [37] evaluated the absorption of natural progesterone from alcoholic gel-based transdermal formulations in vitro and in vivo. They have studied the impact of hydrophilic surfactants (polyoxyethylene (7) oleylether (Oleth-7), polyoxyethylene (10) oleylether (Oleth-10), polyoxyethylene (20) oleylether (Oleth-20), polyoxyethylene (20) cetylether (Ceteth-20), polyoxyethylene (20) stearylether (Steareth-20) and polyoxyethylene (20) behenylether (Beheneth-20) and isopropyl myristate (IPM), benzyl alcohol (BA) or propylene glycol dicaprylate (PGDC) on the penetration of Prog through rat skin. The optimal carrier for Prog was an ethanolic gel containing Oleth-20 and PGDC. The formulation demonstrated high transdermal absorption in vitro and in vivo. Plasma concentration of progesterone after repeated-dose transdermal application was 13.9 ± 4.85 ng/mL (*p* < 0.01) after 48 h.

The subject of the study by Szcześniak et al. [47] was the analysis of the effect of selected absorption promoters (N,N-dimethylacetamide (DMA), propylene glycol-1,2, ethanol 760 g/L and Tween 20) contained in Carbopol 934 P on HC permeation. The authors found that increasing the concentration of uptake promoters increased the amount of HC released. The value of the constant release rate increases in the presence of ethanol, Tween 20 and DMA.

### 5.2. Sulfoxides

Dimethylsulphoxide (DMSO) is a frequently used penetration enhancer as a ‘universal aprotic solvent’. Although it shows high efficiency in penetrating hydrophilic and lipophilic substances, it is problematic to use. In high concentrations (>60%), it can cause erythema and wheals of the stratum corneum and may denature some proteins [116]. Application of 90% DMSO to the skin of healthy volunteers caused erythema, scaling, contact urticaria, stinging and burning sensations and systemic symptoms [119]. Irreversible skin damage can also be caused by the chemically related materials dimethylacetamide (DMAC) and dimethylformamide (DMF) [120]. In contrast, decylmethyl sulphoxide (DCMS) acts reversibly on human skin and is a potent enhancer for hydrophilic permeants [116].

A hydrogel based on hydroxypropyl methylcellulose (HPMC) containing ethanol (25% *w*/*w*) as a potential substrate for TS was proposed by Heo et al. [32]. The effectiveness of hydrogels modified with the addition of absorption promoters (propylene glycol, butylene glycol, diethanolamine, dimethyl sulfoxide/DMSO, N-methyl pyrrolidone/NMP) was studied in a rat model in vivo and in vitro using hairless mouse skin. The authors achieved a significantly high TS plasma concentration profile using the developed hydrogel substrates. The combination of diethanolamine (2%) and NMP (6%) was the most effective among tested absorption promoters.

### 5.3. Alcohols, Glycols and Glycol Ethers

Alcohols are among the most commonly used sorption promoters. Ethanol increases the permeation of both polar and non-polar molecules. Depending on the concentration of the ethanol used in the donor solution/formulation and on the lipophilicity of drug/actives, different mechanisms of action are proposed. Ethanol at a concentration of 25% interacts with polar lipid groups causing fluidisation of the lipid bilayer. In contrast, ethanol at concentrations >50% causes conformational changes of α-keratin and partial extraction from the lipid bilayer matrix [121,122]. The permeation rate of ethanol through human skin is 1 mg/cm^2^/h [123]. Alcohols are very good solvents and solubilisers. Unfortunately, they evaporate quickly and cause dryness of the skin [124]. Propylene glycols are effective cosolvents. Their action is based on improving drug partition properties and reducing drug-tissue binding by the solvation of α-keratin. In addition, they affect lipids in the stratum corneum [125,126]. It has been suggested that propylene glycol shows optimum activity in a system with oleic acid and in a propylene glycol-isopropyl alcohol (30:70% (*v*/*v*) system with essential oil [127].

Pabla et al. [29] proposed modifying the composition of commercially available transdermal hydroalcoholic gels containing 1% testosterone (Androgel^®^, Testim^®^ and the generic form). They replaced part of the ethanol with isopropyl alcohol (IPA). The effectiveness of the modified hydrogel based on Carbopol Ultrez 10 was tested using in vitro release/permeation experiments versus Androgel^®^. The study confirmed that IPA does not increase the bioavailability of testosterone from hydroalcoholic gel preparations. This may be due to a potential interaction of TS–Carbopol Ultrez 10. The authors suggest that IPA may enhance the release of TS from other types of Carbopol. The strongly dehydrating nature of ethanol causes rapid drying of the epidermis, making it difficult for API to penetrate the skin. The less volatile isopropyl alcohol prevents this process while maintaining an optimum ethanol concentration gradient for efficient hormone permeation. IPA also exhibits good cosolvent properties, without affecting the consistency or aesthetics of the finished formulation.

Antares Pharma has developed and patented Advanced Transdermal Delivery (ATD™) technology, which is based on a combination of solvents and compounds that enhance the permeation of API through the skin. The advantage of this technology is the possibility to optimise physicochemical parameters of the preparation (rheological properties, pH) and to modulate hormone permeation through the skin (selection of concentrations of active substance and excipients, the thermodynamic activity of the molecule in the substrate). The most commonly used solvents in ATD™ technology are alcohols, glycols and glycol ethers. These compounds have a synergistic effect on delaying the crystallisation of the drug (while maintaining its molecular form), which enables the skin permeation of APIs (also lipophilic drugs) [61]. Olsson et al. [33] compared the rate of transdermal transport of testosterone from hydrogel 1% and 2% vs. Testogel. They performed the study on a Caucasian male model with reduced blood testosterone levels. A hydrogel (Carbopol 980) based on ATD™ was used as a TS carrier, which influenced faster testosterone absorption, according to first-order kinetics. The blood TS concentration profile was similar to the circadian one.

### 5.4. Esters

Sucrose esters are frequently used surfactant compounds. The properties of these compounds depend on fatty acid esterification and the nature of esterified fatty acid molecules in the sucrose [128]. Sucrose laurate increases the penetration of poorly water-soluble drugs [112]. Isopropyl myristate is a lipophilic molecule and can liquefy the lipids of the stratum corneum intercellular membrane [129].

Vermeire et al. [41], on the other hand, studied the efficacy of sucrose laurate (5%, 15% *w*/*w*) in the skin permeation of estradiol (ES). The study was performed in a male rabbit model and evaluated the absolute bioavailability of the hormone and the skin irritation after single and multiple applications. Two estradiol hydrogels based on hydroxypropyl methylcellulose, differing in laurate sucrose content, were developed and compared with the reference formulation Oestrogel. The base of the original formulation was carbopol 940 with ethanol (30%). It was found that sucrose laurate showed stability in the studied hydrogels during a four-month storage period (7 ± 2 °C). The preparation containing 15% sucrose laurate was characterised by higher bioavailability of ES after a single application. Oestrogel showed a higher efficacy when administered several times. The result of histological examination confirmed a significant increase in skinfold thickness after administration of the 15% sucrose laurate gel (indicating some skin irritation potential). Most surfactants hydrate the skin and an increase in hydration correlate with increased skin permeability. However, in this case, the increased skin penetration is due to sucrose laurate’s ability to disrupt the ability of SC lipids, and consequently to dissolve and extract lipids.

A working group around Zidan [30] investigated the effect of isopropyl myristate (IPM) in hydroalcoholic carbopol gel on the permeation of testosterone through excised human cadaver skin. IPM could change the SC microstructure by fitting into the lipid lamellae because of its hydrophobic nature or could also liquefy the SC lipids because of its branched structure. The formulations tested contained IPM at concentrations of 0–3% *w*/*w*. The hydrogel formulation was supplemented with 73.5% (*w*/*w*) ethanol. A low concentration of ethanol influences increases drug diffusivity by interaction with SC. Moderate concentrations of ethanol increase both the diffusivity and solubility of drugs. Ethanol and IPM showed synergistic effects. The highest TS release was observed with IPM at a concentration of 2% *w*/*w*.

### 5.5. Terpenes

Terpenes enhance the permeation of lipophilic substances, hydrophilic substances and compounds in ionic form. Their advantages are reversible alteration in the stratum corneum, percutaneous absorption enhancement, low toxicity and low irritational effect [112]. When used in low concentrations (1–5%), they show high percutaneous enhancement abilities and low cutaneous irritancy [130]. Their effect depends on chemical structure and physicochemical properties, such as its lipophilicity, size and chirality, boiling point and energy of vaporisation and degree of unsaturation. Their mechanism of action involves temporary accumulation in the stratum corneum (and/or keratin) and disruption of the ordered intercellular lipid system of the stratum corneum [131]. Terpenes have been found to be skin safe and non-irritating. The optimal terpene enhancer is hydrophobic, it is liquid at room temperature, it contains an ester or aldehyde functional group and it is either a triterpene or a tetraterpene [132]. Terpenes show synergism of action with ethanol in the fluidisation of the intercellular lipids [133].

El-Kattan et al. [45] studied in vitro the rate of transdermal transport of Hydrocortisone (HC). The hormone carrier was HPMC gels containing terpenes with different values of lipophilicity log *p* = 1.06–5.36. The authors found a positive correlation between the lipophilicity of the terpenes and the cumulative amount of hydrocortisone permeating through skin. Nerolidol, whose lipophilicity was the highest (log *p* = 5.36 ± 0.38), provided the greatest enhancement for HC flux (35.3-fold over control). Fenchone (log *p* = 2.13 ± 0.30) exhibited the lowest enhancement of HC flux (10.1-fold over control). The higher enhancement activity of hydrocarbon terpenes can be attributed to their higher thermodynamic activity in the hydrogel.

In another study [35], the authors compared the rate of transdermal testosterone transport from hydrogels containing propylene glycol or limonene or oleic acid or transcutol or a combination of two permeation enhancers, respectively. They found that the highest amount of TS was released from hydrogel, which contains limonene and propylene glycol (in the concentration of 15%).

Monti et al. [42] to promote permeation of estradiol through the skin chose six oils: cajuput, cardamom, melissa, myrtle, niaouli and orange oil, all used at the 10% *w*/*w* concentration in propylene glycol (PG). Tests were performed in hairless mouse skin in vitro model. The results show that propylene glycol has a synergistic effect with the terpenes in the organogels increasing the penetration of the hormone. In addition, 1.0% NIA (main terpene components: 1,8-cineole, α-pinene, α-terpineol, D-limonene) significantly increased the estradiol transdermal flux. It has been suggested that essential oils may disrupt the ordered arrangement of lipids in the SC or increase the solubility of API diffusing into the stratum corneum [134,135].

### 5.6. Ureas and Lactam

Urea has an emollient and moisturising effect [136]. It lowers the stratum corneum barrier by changing its hydration. In concentrations of 10–50%, urea has a keratolytic effect (loosens keratin connections). It has the ability to the formation of hydrophilic diffusion channels within the epidermal barrier [116,136,137]. The disadvantage of urea is its ability to increase the water content of the stratum corneum (which acts as a humectant) and preserve its fluidity [112]. Laurocapram reduces the diffusional resistance of a substance into the stratum corneum and inserts it into the lipid bilayer region [138]. It has been found that it can increase the permeation of hydrophilic compounds, hydrophobic compounds and peptides [139,140].

Bentley et al. [44] investigated the effect of a poloxamer 407 base containing lecithin or urea on the dermal penetration of hydrocortisone acetate (HCA, an analog of the natural glucocorticosteroid produced in the adrenal cortex). Tests were conducted in an in vitro model through hairless mouse skin. The transdermal transport of the hormone followed first-order kinetics. Diffusion and retention of HCA in the skin depended on the concentration of the absorption promoters used. Lecithin at a concentration of 8.0% (*w*/*w*) caused retention of seven times more than that of urea at a concentration of 12.0% (*w*/*w*). Lecithin affects the stratum corneum lipid matrix causing disruption of the intercellular lipid lamellar structure. HCA is a lipophilic drug and lecithin deposits the HCA in the skin layers. Urea increases the SC hydration and causes an exfoliative effect. The optimal formulation showed the characteristics desired by the authors: maximum retention of HCA in the skin and its minimal systemic absorption.

Currently, there is no optimal dermatological preparation containing progesterone on the market because it is metabolised by 5-α-reductase in the skin [141]. However, ready-to-use hydrogel-based vaginal formulations of progesterone are available (Table 2). Research is currently underway to develop a hydrogel preparation that will ensure the stability of Prog during its passage through the skin [142]. The subject of analysis by Valenta et al. [35] was the evaluation of the effect of permeation enhancers such as propylene glycol, urea and laurocapram on the percutaneous absorption of progesterone (Prog) from carbopol hydroalcoholic gels. The study was performed in vitro, in hairless rat skin or ears of female pigs model. The most effective promoter of absorption was 10% laurocapram.

### 5.7. Permeation Enhancement Technologies

In recent years, substances/mixtures of substances have been patented for their suitability in formulating hydrogels containing protein and peptide hormones. The pharmaceutical formulation Testim™ (testosterone), patented by Bentley Pharmaceuticals contains CPE-215^®^ (cyclopentadecanolide). This substance supports the transport of proteins, peptides and low-molecular drugs across natural membranes into the bloodstream. SEPA^®^ (1,3-dioxolanes) increases skin absorption by liquefying lipids in the outer layer of the skin. NexACT^®^ (alkyl-2-[substituted amino]-alkanoate ester, alkanol alkanoate enables fast and efficient dermal absorption of APIs [61]. On the other hand, Ferring Pharmaceuticals Ltd. developed Testavan^®^ using F.A.S.T. (Ferring’s Advanced Skin Technology). The formulation includes ethanol, propyleneglycol and diethylenglycolmonoethylether, which increase the bioavailability of testosterone through the skin. In addition, the formulation is applied to the skin using a hands-free applicator, which reduces the risk of secondary transfer of testosterone to other parts of the body/person [97].

Another study [40] investigated the effect of albumin (Alb) added to glycerol hydrogel on the permeation of corticotropin (ACTH). It was found that, depending on the amount of albumin used, it can delay or increase the hormone release process. The highest efficacy was obtained using Alb at a concentration of 15 mg/g in a 1:1 ratio to ACTH. Albumin can influence the increased transdermal absorption of ACTH.

## 6. Mechanism of Penetration of Hormones through the Skin

**Figure 2:**
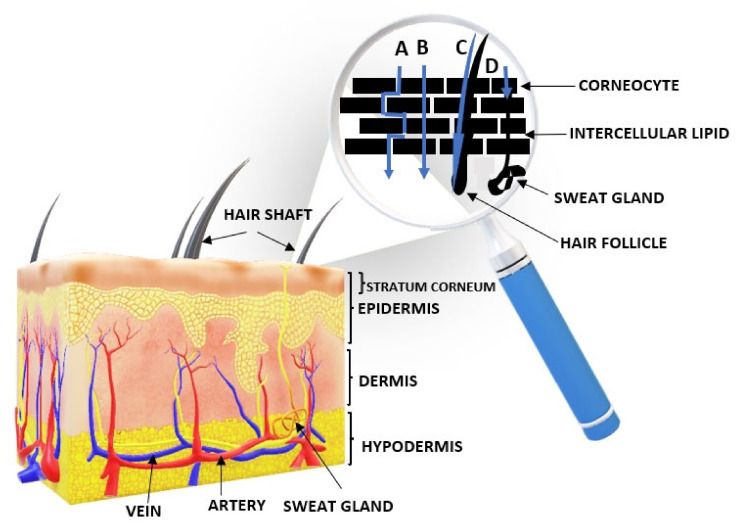
Structure of the skin. Routes of drug permeation across the stratum corneum: A—intercellular penetration pathway; B—transcellular penetration pathway; C—transfollicular drug delivery; D—transglandular drug delivery.

Hydrogels of steroid hormones (progesterone, testosterone, oestradiol, hydrocortisone/Figure 1), which belong to non-polar, low molecular weight compounds, are commercially available [143,144]. The substrate for the synthesis of steroid hormones is cholesterol [145]. Steroid hormones (like cholesterol) exhibit hydrophobic properties (they do not dissolve in water), which determines the route of their penetration through the skin (Figure 2) [72]. After topical application, the hormone is released from the substrate, then diffuses into the stratum corneum via the mainly lipid intercellular pathway [73]. In the next step, to reach the target cell, the API must bind to transport proteins (90% of plasma is water). This has the effect of prolonging the half-life of the hormone [146]. The transfollicular pathway is of little relevance, as the skin appendages occupy less than 0.1% of the skin surface. In addition, API diffusion may be hindered by the secretory activity of the sebaceous and sweat glands [147].

A different mechanism of hormone absorption through the skin is observed in the case of a dermatological condition. The skin lesions cause a disruption of the stratum corneum structure, which alters the permeability of the skin and impairs its barrier function [148]. Ostróżka-Cieślik and co-workers [39] developed insulin hydrogels for the potential treatment of the diabetic foot. It can be assumed that insulin (a macromolecular and water-soluble compound, like most peptide hormones) will diffuse through the damaged skin and then permeate into the general circulation. It should be noted that, as a result of the action of plasma proteases, the hormone is degraded and has a shorter half-life than steroid or thyroid hormones [70]. It is worth mentioning that the base designed to be applied to the wound surface administration must be sterile. They can be sterilised in an autoclave, but there is a risk of changing their viscosity [149].

It is important to bear in mind that topically applied hormones may act on specific receptors located in skin cells. Receptors for peptide hormones are predominantly located on the cell surface, whereas receptors for steroid hormones are located in the cytoplasm or nuclear compartments [64,150]. Sex hormone receptors are expressed in epidermal keratinocytes, affecting epidermal barrier homeostasis [151,152,153]. Steroid hormones can modify the structure of the lipid layer. Estradiol increases lipid mobility on the plasma membrane, while progesterone decreased it [154]. Peptide hormones show pleiotropic effects on the skin [64].

## 7. Conclusions and Future Perspectives

Advances in molecular biology and biotechnology have made it possible to obtain a wide range of biologically active substances, including hormones. However, the difficulty is to develop carriers for them that will effectively deliver the drug to the site of action. The development of dermal drug forms for hormones is one of the intensely developing research areas. Hydrogels are an innovative alternative to conventional forms of dermatological drugs (i.e., ointments, creams, emulsions), although the number of hydrogels containing hormones available on the market is small. Data from trials on ClinicalTrials.gov suggest that several formulations are in clinical trials e.g., Androgel (testosterone gel), FE 999303 (testosterone gel), COL-1620 (progesterone gel), Crinone (progesterone gel) and estradiol gel. Further research to optimise the composition of hydrogels containing hormones should focus on the aim to increase the transdermal bioavailability of this group of drugs and deliver a higher dose of hormone in a smaller volume of the hydrogel. This would also reduce the risk of interpersonal (to the partner and/or children) transfer of the gel not absorbed at the site of application. From the author’s perspective, attention should be given to the development of hydrogel carriers allowing the administration of more than one drug simultaneously.

## Figures and Tables

**Table 1:** Strategies used to incorporate the hormone into the hydrogel.

Author, Year of Publication	Hormone, Dose	Polymer	Permeation Enhancers	Release Method/Diffusion Cell Type	Skin Model	Studied Time	Effects
Testosterone (TS)							
An et al., 2003 [31]	30 mg/g (3% *w*/*w*)	polyvinyl alcohol, (PVA) with 2% PIB	Dodecylamine, HPE101, oleic acid, lauric acid	in vivo; in vitro/Keshary–Chien permeation cells	rat skin	24 h	Dodecylamine (3%) increased the rate of skin penetration
Heo et al., 2005 [32]	10 mg/g (1% *w*/*w*)	HPMC 2910	Propylene glycol, butylene glycol, diethanolamine, DMSO, NMP	in vivo; in vitro/Keshary-Chien permeation cells	rat skin, hairless mouse skin	8 h	Combination of diethanolamine (2%) and NMP (6%) was the most effective among tested
Pabla et al., 2007 [29]	10 mg/g (1% *w*/*w*)	Carbopol Ultrez 10 (0.9% *w*/*w*)	Isopropyl alcohol (IPA)	in vitro Franz-diffusion cells	hairless guinea pig skin; cellulose ester membrane; CelgardR 2400 membrane	12 h	IPA does not increase the bioavailability of API from hydroalcoholic gel preparations
Olsson et al., 2014 [33]	10 mg/g (1% *w*/*w*), 20 mg/g (2% *w*/*w*)	Carbopol 980	ATD™ (ethanol, propylene glycol, diethylene glycol monoethyl ether)	in vivo	Caucasian men	48 h	ATD™ increase bioavailability of TS vs. Testogel^®^
Zidan et al., 2017 [30]	16,2 mg/g (1.62% *w*/*w*)	Carbopol 980	Isopropyl myristate (IPM)	in vitro Diffusion cells	human cadaver skin	24 h	In the presence of 2% IPM+73.5% ethanol, an 11-fold increase in TS release was observed
Bilal et al., 2018 [34]	10 mg/g (1% *w*/*w*)	Carbopol 980	Propylene glycol, limonene, oleic acid, transcutol	in vitro Vankel enhancer cell	polyvinylidene fluoride membranes (PVDF) 0.22 µm; rat skin	24 h	Limonene and propylene glycol (15%) increase API penetration
Progesterone (Prog)							
Valenta et al., 1997 [35]	30 mg/g (3% *w*/*w*)	Carbopol 940	Propylene glycol, ethanol, laurocapram	in vitro/ Franz-diffusion cells	hairless rat skin, ears of female pigs	24 h	10% laurocapram was to be the most efficient enhancer for Prog from carbopol hydroalcoholic gels
Kählig et al., 2009 [36]	10 mg/g (1% *w*/*w*)	Chitosan-EDTA, carrageenan, chitosan-glycolic acid	-	in vitro/ Franz-diffusion cells	porcine abdominal skin	48 h	Chitosan-glycolic acid can be recommended for a transdermal application of hormone
Matsui et al., 2015 [37]	30 mg/g (3% *w*/*w*)	Carboxyvinylpolymer	PGDC, Oleth-7, Oleth-10, Oleth-20, 1,3-BG, Ceteth-20, Steareth-20, Beheneth20, BA, isopropyl myristate ethanol	in vivo, in vitro/ Franz-diffusion cells	dorsal skin of a 6-week-old male rat or rat abdominal skin	24 h	The Oleth-20 and PGDC have the ability to maintain a high activity of Prog and high diffusivity or solubility of Prog in the epidermis
Bassani et al., 2017 [38]	50 mg/g (5% *w*/*w*)	VersaBase^®^ Gel	-	in vitro/ Franz-diffusion cells	human trunk skin	48 h	Prog in VersaBase^®^Gel is absorption through the skin
Insulin (INS)							
Ostróżka-Cieślik et al., 2021 [39]	1 mg/g (0.1% *w*/*w*)	Carbopol Ultrez 10, Carbopol Ultrez 30, methyl cellulose, glycerol ointment	-	in vitro/ enhancer cell	cellulose dialysis membrane Spectra/Por^®^ 2 (MWCO of 12–14 kDa)	10 h	Methyl cellulose-based hydrogel released API reaching 75% after 9 h
Corticotropin (ACTH)							
Siemiradzka et al., 2021 [40]	15 mg/g (1.5% *w*/*w*), 20 mg/g (2% *w*/*w*)	Glycerol ointment	Albumin	in vitro/enhancer cell or Franz-diffusion cells	cellulose dialysis membrane Spectra/Por^®^ 2 (MWCO of 100 kDa) or porcine ear skin	24 h	Albumin can delay or increase ACTH permeation
β-Estradiol (ES)							
Vermeire et al., 1996 [41]	0.6 mg/g (0.06% *w*/*w*)	Methocel™ K 100M (hydroxypropyl methylcellulose)	Sucrose laurate (5%, 15% *w*/*w*)	in vivo	male rabbits	12 h	Sucrose laurate (15%) showed absorption enhancing properties and has some skin irritation potential.
Monti et al., 2002 [42]	10 mg/g (1% *w*/*w*)	Carbopol 1342	Terpene containing essential oils: cajuput, cardamom, melissa, myrtle, niaouli (NIA), orange	Diffusion cells	hairless mouse skin	30 h	1.0% NIA significantly increased the estradiol transdermal flux.
Barreiro-Iglesias et al., 2003 [43]	40 mg/g (4% *w*/*w*)	Carbopol 934NF	Pluronic F-127, Tween 80, SDS, BkCl	Horizontal diffusion cells	cellulose acetate	not specified	Carbopol/surfactant aggregates: they enhance the solubility of hydrophobic drugs using low-surfactant proportions and they make it possible to control drug release rates.
Hydrocortisone (HC)							
Bentley et al., 1997 [44]	10 mg/g (1% *w*/*w*)	Poloxamer 407	Urea, lecithin	Franz-diffusion cells	hairless mouse skin	24 h	Lecithin in poloxamer gels can increase skin retention of hidrocortisone acetate.
El-Kattan et al., 2000 [45]	20 mg/g (2% *w*/*w*)	Hydroxypropyl methylcellulose (HPMC)	Terpene: terpinen-4-ol, verbenone, fenchone, carvone, menthone, α-terpineol, cineole, geraniol, thymol, cymene, d-limonene, nerolidol	Franz-diffusion cells	hairless mouse skin	24 h	Positive correlation between the lipophilicity of the terpenes and the cumulative amount of hydrocortisone permeating through skin.
Meler et al., 2013 [46]	10 mg/g (1% *w*/*w*)	Methylcellulose, carboxymethylcellulose, Carbopol 934P, chitosan	1,2-propylene glycol	Hanson diffusion chambers	semi-permeable membrane	2.5 h	Prepared gels based on cellulose have a higher rate of diffusion than prepared with Carbopol 934 *p*. Addition of 1% chitosan affects the acceleration of the release.
Szcześniak et al., 2013 [47]	10 mg/g (1% *w*/*w*)	Carbopol 934P	1,2-propylene glycol, N,N-dimethylacetamide, Tween 20, ethanol	Varian VK 7025 dissolution apparatus	semi-permeable membrane	2.5 h	The value of the constant release rate increases in the presence of ethanol, Tween 20, and DMA.

Abbreviations: 1,3-BG, 1,3-butylene glycol; API, active pharmaceutical ingredient; BA, benzyl alcohol; Beheneth20, polyoxyethylene (20) behenylether; BkCl, benzalkonium chloride; Ceteth-20, polyoxyethylene (20) cetylether; DMA, N,N-dimethylacetamide; DMSO, dimethyl sulfoxide; HPE-101, 1-[2-(decylthio)ethyl]azacyclopentan-2-one; HPMC, hydroxypropyl methyl cellulose; NIA, niaouli essential oil; NMP, N-methylpyrrolidone; Oleth-7, Polyoxyethylene (7) oleylether; Oleth-10, polyoxyethylene (10) oleylether; Oleth-20, polyoxyethylene (20) oleylether; PGDC, propylene glycol dicaprylate; PIB, polyisobutylene; SDS, sodium dodecylsulfate; Steareth-20, polyoxyethylene (20) stearylether.

**Table 2:** Physicochemical parameters of hormones and examples of commercial hydrogels containing hormones.

Hormones/ Molecular Weight	Solubility	Partition Coefficient (log Po/w)	Half-Life [min]	Commercial Preparations /Used Hydrogel	Bioavailability of the Hormone	References
Testosterone 288.4 Da	40 mg/L at 37 °C 18–25 mg/L at 20 °C	3.3	10–100	Androgel™ (1% *w*/*w*; AbbVie, Inc.)/Carbopol 980 Androgel 1.62™ (1.62% *w*/*w*, AbbVie, Inc.)/Carbopol 980 Testim™ (1% *w*/*w*, Auxilium Pharmaceuticals)/Carbopol Vogelxo™ (1% *w*/*w*, Upsher-Smith Laboratories)/Carbopol Type B, Carbopol Type C Testogel^®^ (1% *w*/*w*, Bayer Vital Gmbh)/Carbopol 980 Fortesta™ (2% *w*/*w*, Endo Pharmaceuticals)/Carbopol 1382 Fortigel™ (2% *w*/*w*, ProStrakan Ltd.)/Carbopol 1382 Tostrex™ (2% *w*/*w*, ProStrakan Ltd.)/Carbopol 1382 Tostran™ (2% *w*/*w*, ProStrakan Ltd.)/Carbopol 1382 Itnogen™ (2% *w*/*w*, ProStrakan Ltd.)/Carbopol 1382 Testavan^®^ (2% *w*/*w*, Ferring Pharmaceuticals Ltd.)/Carbopol 980	~10–15%	[30,95,96,97]
Progesterone 314.5 Da	Insoluble in water, sparingly soluble in acetone, ethanol: 0.125 g/mL	3.9	5–20	Crinone^®^ (4% *w*/*w* or 8% *w*/*w*, Columbia Laboratories, United Kingdom)/Carbopol 974P (vaginal gel) Prochieve^®^ (8% *w*/*w*, Fleet Laboratories Ltd.)/Carbopol 934P (vaginal gel)	~20%	[96,98]
Estradiol 272.4 Da	0.399 mg/dL at 35 °C	4.01	~60	Divigel (0.1% *w*/*w*, Delfarma, Inpharm, Orion)/Carbopol 974 P Estreva (0.1% *w*/*w*, Theramex)/carboxy-polyvinyl polimer Elestrin (0.06% *w*/*w*, Meda Pharamceuticals)/Carbopol 940 Sandrena^®^ (0.5% *w*/*w*, 1% *w*/*w*, Orion Pharma)/Carbopol 974 P EstroGel^®^ (0.06% *w*/*w*, ASCEND Therapeutics)/Carbopol 934 P Oestrogel (0.06% *w*/*w*, Besins Healthcare)/Carbopol 940	~10%	[53,96,99,100]
Hydrocortisone 362.5 Da	320 mg/L at 25 °C	1.61	~100	Corticool™ (1% *w*/*w*, Tec Labs)/hypromellose Alcortin A (2% *w*/*w*, Novum Pharma, LLC)/Carbopol	NA	[101,102]

## Data Availability

The data presented in this study are available on request from the corresponding author.
